# Acute on Chronic Venous Thromboembolism on Therapeutic Anticoagulation

**DOI:** 10.1155/2013/295261

**Published:** 2013-10-08

**Authors:** Byron Bassi, L. Connor Nickels, F. Eike Flach, Guiliano DePortu, Latha Ganti

**Affiliations:** ^1^Department of Emergency Medicine, University of Florida College of Medicine, 1329 SW 16th Street, P.O. BOX 100186, Gainesville, FL 32610-0186, USA; ^2^Division of Clinical Research, Toral Family Foundation, Center for Brain Injury Research and Education, University of Florida College of Medicine, 1329 SW 16th Street, P.O. BOX 100186, Gainesville, FL 32610-0186, USA

## Abstract

A case of proximal venous thromboembolism in a patient who presented to the ED with lower extremity pain is presented. Making this diagnosis is very important as fifty percent of patients with symptomatic proximal DVTs will go on to develop PE without treatment. This report underscores the utility of bedside ultrasonography in the emergency department.

## 1. Introduction

Venous thromboembolic disease is fairly common, with an approximate yearly incidence exceeding one in every 1000 adults [1], and two-thirds of these will present as isolated deep vein thrombosis (DVT) [2]. While more of these patients will have distal rather than proximal DVT, the mortality rate of proximal DVT is almost double that of distal DVT due to its propensity to migrate to the lungs and cause acute pulmonary embolus (PE) [3]. Multiple characteristics have been looked at in an attempt to differentiate acute from chronic DVT, as these are treated very differently. It can be difficult to differentiate acute from chronic DVT with ultrasound alone [4]. However, lumen echogenicity and vessel elasticity are two characteristics that have shown promise in aiding with this difficult diagnosis [5, 6], as chronic thrombi are more echogenic and less elastic than acute thrombi [7, 8].

## 2. Case

A 40-year-old male presented to the emergency department with the complaint of left lower extremity pain and swelling for three weeks which had acutely worsened. His past medical history was significant for PE and DVT, most recently five months prior to presentation. He was on daily Coumadin but had difficulty consistently maintaining a therapeutic INR. His most recent INR was 3.9 three days prior to admission. He had been instructed by his primary care physician to hold Coumadin for two days and then restart, which he did the day prior to presentation. Physical exam revealed a warm, erythematous left lower extremity. He was tender to palpation of the calf and had 2+ pitting edema distally from his knee. Distal pulses of his left leg were intact, and he had full strength and range of motion of the knee and ankle.

A high frequency 7.5–10 MHz linear array transducer was used to perform the lower extremity ultrasound. Standard, water-soluble ultrasound gel was applied to the patient's groin. The femoral region was scanned in the transverse plane, proximally from the level of the common femoral vein (CFV) just proximal to the junction of the long saphenous vein, distally through the division of the superficial and deep femoral veins. The vein was compressed every 2-3 cm in the usual fashion. The ultrasound demonstrated full compressibility of the proximal segment of the common femoral vein, with loss of coaptation distally from the division of the superficial and deep femoral arteries. Additionally, echogenic material was seen within the vessel lumen in the distal portion of the superficial femoral vein and was not seen more proximally though the vessel did not completely collapse. The patient's INR was found to be subtherapeutic at 1.5. Ultrasound examination of the right lower extremity demonstrated full compressibility of the veins. Given the acute exacerbation of the patient's symptoms and the lack of echogenic material within the proximal vessel lumen, he was started on heparin infusion for treatment of presumed acute-on-chronic DVT. The patient was admitted to medicine, and a full venous duplex bilateral lower extremity ultrasound was performed by radiology, demonstrating occlusion of the left superficial femoral vein extending through the popliteal vein with partial thrombosis within the common femoral vein. He was transitioned from heparin to Lovenox as a bridge for his subtherapeutic INR and subsequently discharged home after an uncomplicated hospital stay.

## 3. Discussion

Proximal DVT is a potentially devastating disease. Accurate and early diagnosis is vitally important as fifty percent of patients with symptomatic proximal DVTs will go on to develop PE without treatment [9]. Even with a high level of clinical suspicion, a definitive diagnostic test is required since most classical signs and symptoms of the disease are poorly predictive of the diagnosis [10], and treatment itself carries a number of complications [11]. 

Ultrasound was first used to diagnose DVT over thirty years ago, [12] and while contrast venography remains the gold standard for diagnosis of DVT, compression ultrasonography has nearly equivalent diagnostic accuracy and has become the diagnostic test of choice [13]. As bedside ultrasound became more and more common, emergency physicians began using two-point compression ultrasonography as a fast and accurate method of diagnosing DVT [14]. Moreover, sensitivity and specificity of 100% and 99%, respectively, can be achieved with as little as ten minutes of training [15]. 

However, compression ultrasonography is not without limitations and has been shown to be less reliable in patients with recurrent DVT because as many as 50% of scans can still be abnormal one year after the initial DVT [16]. It has been shown in this patient population that noncompressibility of previously normal veins or an increase in abnormal vein diameter can be used to diagnose recurrent DVT [17]. Neither of these methods have been looked at for use by emergency physicians. Additionally, acquiring old ultrasound images can be time consuming or simply not feasible. In the case of our patient, no previous ultrasound studies were available for comparison.

**Figure 1:**
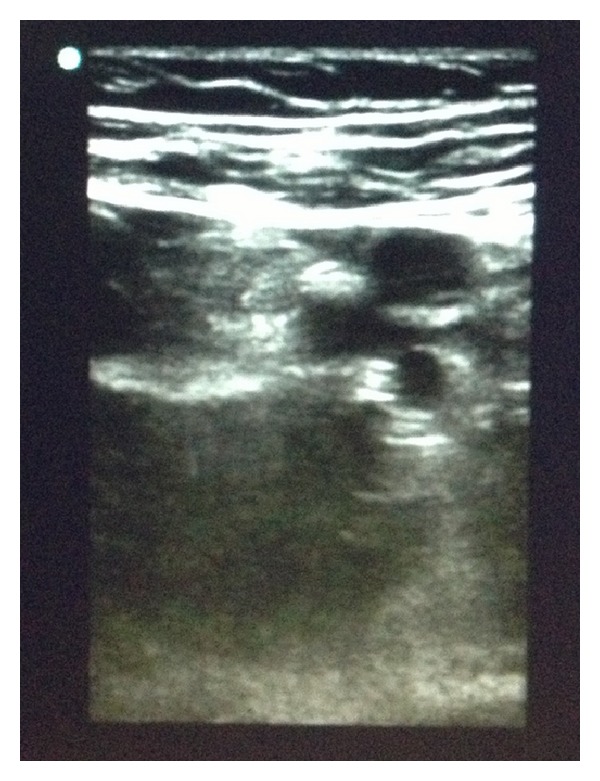
Compression of the common femoral vein at the level of the bifurcation of the common femoral artery.

**Figure 2:**
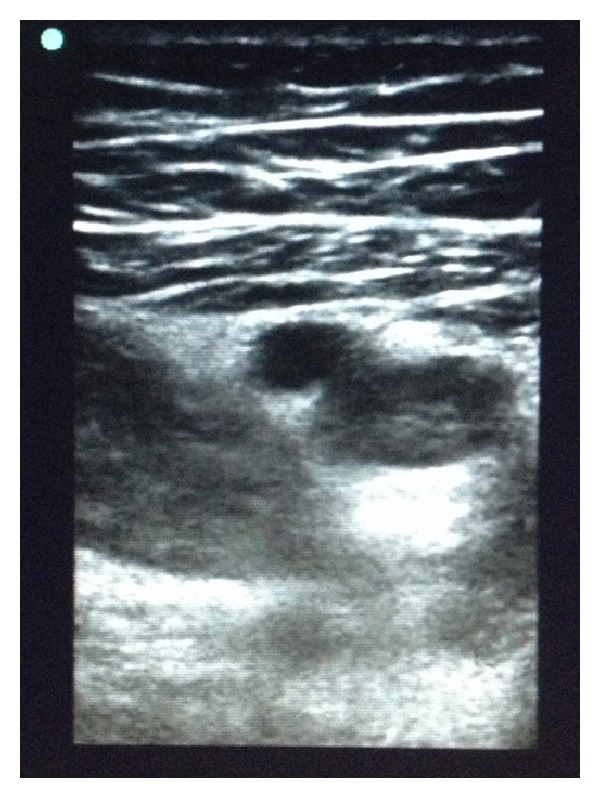
Compression of the deep femoral vein.

It has been shown that thrombus echogenicity can be used to differentiate acute and chronic thrombi [5] and that vessel elasticity is at least as accurate at discriminating acute and chronic thrombi[6]. However, both of these methods require postscan data analyses and complex mathematical calculations that currently cannot be done at the bedside. Knowing the characteristics that can differentiate acute from chronic thrombi, we made an educated assumption about our patient. The lumen of the common femoral vein appeared to be the same echogenicity of the superficial and deep femoral arteries adjacent to it (Figure 1) while more distally the vein was clearly more echogenic (Figure 2). Additionally, the proximal portion of vein deformed much more than the more distal segment. While we had no formal calculations of echogenicity or elasticity and no prior study to compare with, we expected that if the entire thrombus was the same age it would remodel at a similar rate throughout and therefore show similar echogenicity and elasticity proximally as well as distally. Based on this, we felt confident diagnosing an acute-on-chronic DVT. Further investigation is needed to determine whether an “eye test” that combines the multiple characteristics differentiating acute and chronic DVTs can accurately diagnose DVT in this patient population or whether the postimaging analyses discussed above can be done in real time while scanning. If so, emergency physicians will have yet another powerful tool at their disposal when using bedside ultrasound.
